# Supplementary material for: Perioral secretions enable complex social signaling in African mole-rats (genus Fukomys)
Source: Sci Rep. 2022 Dec 26;12:22366. doi: 10.1038/s41598-022-26351-3 (PMC9792591; doi:10.1038/s41598-022-26351-3)
Supplement: Supplementary file 1 — Supplementary Legends. [file 41598_2022_26351_MOESM1_ESM.docx]

**Supplementary Files**

**Supplementary Figure 1:** Histology of perioral glands in the naked mole-rat (*Heterocephalus glaber*). A: male. B: female.

**Supplementary Figure 2:** A: Gaussian modeling of cereal (here denoted as “food”) and hay volatiles derived from the mole-rats’ regular food, only true positives under the green line were selected. B: Intersection plot showing that a similar number of compounds is found in hay plus food and in mole rats while only ~10 % (*n* = 68) is shared between all the three sets.

**Supplementary Table 1:** Information on histologically examined mole-rats.

**Supplementary Table 2:** Information on mole-rats examined to score perioral stain patterns.

**Supplementary Table 3:** Results from olfactory preference tests in Micklems mole-rats (*Fukomys micklemi*).

**Supplementary Table 4:** Information on giant mole-rats (*Fukomys mechowii*) sampled for GCxGC-MS.

**Supplementary Table 5:** Information on volatiles derived from giant mole-rat perioral sebum.

**Supplementary Table 6:** Information on volatiles derived from the mole-rats’ hay and cereal food.
