# Supplementary figures and images for: Perioral secretions enable complex social signaling in African mole-rats (genus Fukomys)
Source: Sci Rep. 2022 Dec 26;12:22366. doi: 10.1038/s41598-022-26351-3 (PMC9792591; doi:10.1038/s41598-022-26351-3)

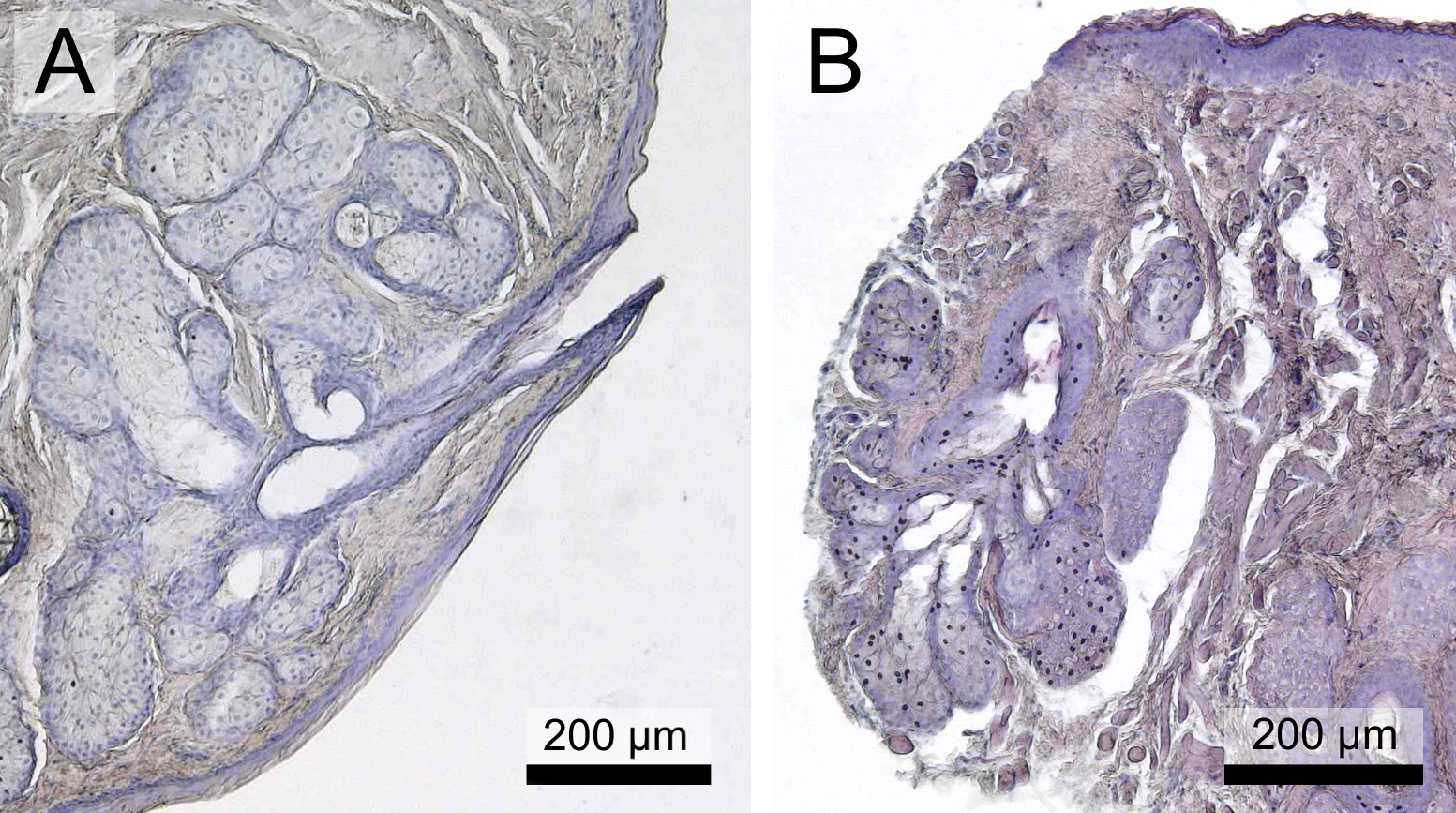

Supplement: Supplementary file 2 — Supplementary Figure 1. [file 41598_2022_26351_MOESM2_ESM.tiff]

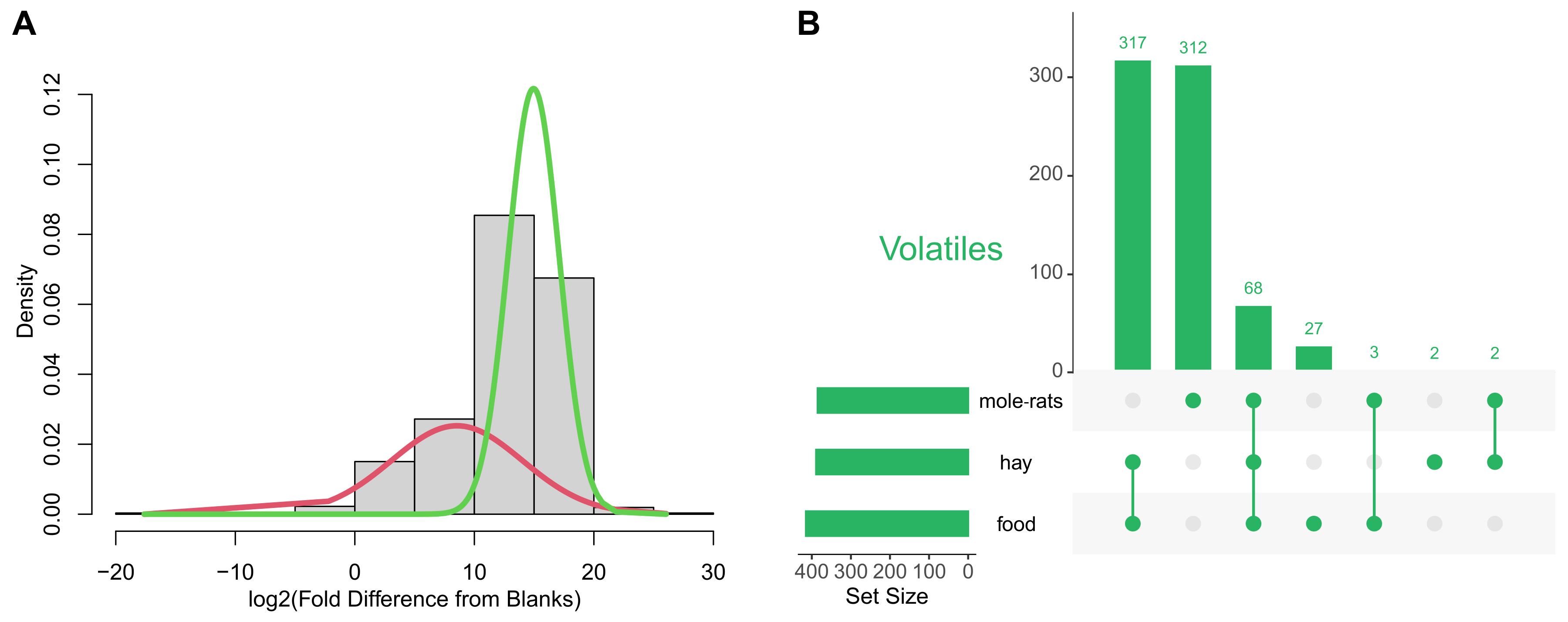

Supplement: Supplementary file 3 — Supplementary Figure 2. [file 41598_2022_26351_MOESM3_ESM.tiff]
